# Association of IGF-1 Level with Low Bone Mass in Young Patients with Cushing's Disease

**DOI:** 10.1155/2023/3334982

**Published:** 2023-07-04

**Authors:** Wanwan Sun, Quanya Sun, Qiaoli Cui, Min He, Wei Wu, Yiming Li, Hongying Ye, Shuo Zhang

**Affiliations:** Department of Endocrinology and Metabolism, Huashan Hospital, Fudan University, Shanghai 200040, China

## Abstract

**Purpose:**

Few related factors of low bone mass in Cushing's disease (CD) have been identified so far, and relevant sufficient powered studies in CD patients are rare. On account of the scarcity of data, we performed a well-powered study to identify related factors associated with low bone mass in young CD patients.

**Methods:**

This retrospective study included 153 CD patients (33 males and 120 females, under the age of 50 for men and premenopausal women). Bone mineral density (BMD) of the left hip and lumbar spine was measured by dual energy X-ray absorptiometry (DEXA). In this study, low bone mass was defined when the Z score was −2.0 or lower.

**Results:**

Among those CD patients, low bone mass occurred in 74 patients (48.37%). Compared to patients with normal BMD, those patients with low bone mass had a higher level of serum cortisol at midnight (22.31 (17.95-29.62) vs. 17.80 (13.75-22.77), *p*=0.0006), testosterone in women (2.10 (1.33–2.89) vs. 1.54 (0.97–2.05), *p*=0.0012), higher portion of male (32.43% vs. 11.54%, *p*=0.0016) as well as hypertension (76.12% vs. 51.67%, *p*=0.0075), and lower IGF-1 index (0.59 (0.43–0.76) vs. 0.79 (0.60–1.02), *p*=0.0001). The Z score was positively associated with the IGF-1 index in both the lumbar spine (*r* = 0.35153, *p* < 0.0001) and the femoral neck (*r* = 0.24418, *p*=0.0057). The Z score in the femoral neck was negatively associated with osteocalcin (*r* = −0.22744, *p*=0.0229). Compared to the lowest tertile of the IGF-1 index (<0.5563), the patients with the highest tertile of the IGF-1 index (≥0.7993) had a lower prevalence of low bone mass (95% CI 0.02 (0.001–0.50), *p*=0.0002), even after adjusting for confounders such as age, gender, duration, BMI, hypertension, serum cortisol at midnight, PTH, and osteocalcin.

**Conclusions:**

The higher IGF-1 index was independently associated with lower prevalence of low bone mass in young CD patients, and IGF-1 might play an important role in the pathogenesis of CD-caused low bone mass.

## 1. Introduction

Cushing's disease (CD), caused by an adrenocorticotropic hormone (ACTH)-secreting pituitary tumor, is a rare disease with approximately 1.2 to 2.4 new cases per million people each year [1].

Osteoporosis has been recognized as a serious consequence of endogenous hypercortisolism since the first description in 1932 [2]. The prevalence of osteoporosis is around 38–50%, and the rate of atraumatic compression fractures is 15.8% in CD patients [3]. After cortisol normalization and appropriate treatment, recovery of the bone impairment occurs slowly (6–9 years) and partially [4, 5]. Hypercortisolemia impairs bone quality through multiple mechanisms [6]. Growth hormone (GH) and insulin-like growth factor 1 (IGF-1) play a crucial role in bone growth and development [7]. IGF-1 is considered essential for the longitudinal growth of bone, skeletal maturity, and bone mass acquisition not only during growth but also in the maintenance of bone in adults [8]. Previous research studies revealed that low serum IGF-1 levels were associated with a 40% increased risk of fractures [9, 10], and serum IGF-1 levels could be clinically useful for evaluating the risk of spinal fractures [11]. In Marl Hotta's research, extremely low or no response of plasma GH to recombinant human growth hormone (hGRH) injection was noted in CD patients. This result suggested that the diminished hGRH-induced GH secretion in patients with Cushing's syndrome might be caused by the prolonged period of hypercortisolemia [12]. Other surveys indicated that glucocorticoids, suppressing GH–IGF-1 and the hypothalamic-pituitary-gonadal axes, lead to decreased number and dysfunction of osteoblast [13].

However, the exact mechanism is still unclear, and few risk factors for osteoporosis in CD have been identified so far. Until now, relevant and sufficiently powered studies in CD patients have been rare [14, 15]. Early recognition of the changes in bone mass in CD patients contributes to early diagnosis of bone mass loss and prompt treatment, which could help minimize the incidence of adverse events such as fractures.

On account of the scarcity of data and pressing open questions concerning risk evaluation and management of osteoporosis, we performed a well-powered study to identify the related factors associated with low bone mass in young CD patients at the time of diagnosis.

## 2. Materials and Methods

### 2.1. Subjects

This retrospective study enrolled 153 CD patients (33 males and 120 females) from the Department of Endocrinology and Metabolism of Huashan Hospital between January 2010 and February 2021. All subjects were evaluated by the same group of endocrinologists for detailed clinical evaluation. This study, which was in complete adherence to the Declaration of Helsinki, was approved by the Human Investigation Ethics Committee at Huashan Hospital, Fudan University (No. 2017M011). We collected data on demographic characteristics, laboratory tests, and bone mineral density.

Inclusion criteria included the following: (1) willingness to participate in the study; (2) premenopausal women ≥18 years old, men ≥18 years old but younger than 50 years old, and young women (<50 years old) with menstrual abnormalities who were associated with CD after excluding menstrual abnormalities caused by other causes; (3) diagnosis of CD according to the updated diagnostic criteria [16]; and (4) pathological confirmation after transsphenoidal surgery (positive immunochemistry staining with ACTH). Exclusion criteria included Cushing's syndrome other than pituitary origin.

### 2.2. Clinical and Biochemical Methods

IGF-1 was measured using the Immulite 2000 enzyme-labeled chemiluminescent assay (Siemens Healthcare Diagnostic, Surrey, UK). Other endocrine hormones, including cortisol (F), 24-hour urinary free cortisol (24hUFC), adrenocorticotropic hormone (ACTH), prolactin (PRL), luteinizing hormone (LH), follicle stimulating hormone (FSH), estrogen (E2), progesterone (P), testosterone (T), thyroid stimulating hormone (TSH), and free thyroxine (FT4), were carried out by the chemiluminescence assay (Advia Centaur CP). Intra-assay and interassay coefficients of variation were less than 8 and 10%, respectively, for the estimation of all hormones.

Bone metabolism markers included osteocalcin (OC), type I procollagen amino-terminal peptide (P1NP), parathyroid hormone (PTH), and 25-hydroxyvitamin D (25(OH)VD), measured in a Roche Cobas e411 analyzer using immunometric assays (Roche Diagnostics, Indianapolis, IN, USA).

The IGF-1 index was defined as the ratio of the measured value to the respective upper limit of the reference range for age and sex. Body mass index (BMI) was calculated using the following formula: weight (kg)/height^2^ (m^2^). The bone mineral density (BMD) measuring instrument was Discovery type W dual energy X-ray absorptiometry from the American HOLOGIC company. Quality control tests were conducted every working day. Before examination, the date of birth, height, weight, and menopause date of the examiner were accurately recorded, and then BMD (g/cm^2^) of the left hip and lumbar spine were measured by DEXA. *Z* value was used for premenopausal women and men younger than 50 years old, and *Z*-value = (measured value − mean bone mineral density of peers)/standard deviation of BMD of peers [17, 18]. In this study, low bone mass was defined as a *Z*-value of −2.0 or lower.

### 2.3. Statistical Analysis

The baseline characteristics were compared between CD patients with and without low bone mass by using the Student's *t*-test for continuous variables and the *χ*^2^ test for category variables. Bone turnover markers, alanine aminotransferase (ALT), triglyceride (TG), IGF-1 index, thyroid stimulating hormone (TSH), free triiodothyronine (FT3), free thyroxine (FT4), testosterone (T), 24 hours of urine cortisol (24 h UFC), and serum cortisol at 8 a.m. (F8 am) and at midnight (F24 pm) were not in normal distribution, so variables mentioned above were Log10-transformed, which could be used as continuous variables during statistical analysis. Participants were categorized into three groups according to tertiles of the IGF-1 index: <0.5986, 0.5986–0.8380, and >0.8380. The linear trend across IGF-1 index tertiles was tested using linear regression analysis for continuous variables and the Cochran–Armitage test for categorical variables. We used a multivariate logistic regression model to identify related factors that are independently associated with the risk of low bone mass. Variables included in the multivariate logistic regression model were selected based on the Spearman rank correlation analysis and established traditional low bone mass risk factors as priors. The results were presented as odds ratios (OR) and the corresponding 95% confidence intervals (CI). Significance tests were two-tailed, with *P* value <0.05 considered statistically significant for all analyses. Statistical analysis was performed using SAS version 9.3 (SAS Institute Inc, Cary, NC, USA).

## 3. Results

### 3.1. The Prevalence of Low Bone Mass in Young Cushing's Disease Patients

From the inpatient system of Huashan hospital, a total of 153 CD patients under the age of 50 for men and premenopausal women (some with menstrual abnormalities were associated with CD) were included, aged from 13 to 49 years, with an average age of 34.25 ± 8.39 years. There were 33 males (21.57%) and 120 females (78.43%). These CD patients included newly diagnosed CD, recurrences of CD, and CD without remission after treatment. There were no differences in the prevalence of different statuses of CD between the two groups (Table 1).

Among these CD patients, low bone mass occurred in 74 patients (48.37%), including 24 men and 50 women. The prevalence of low bone mass was 41.67% and 72.73% in female and male CD patients, respectively, and 42 (56.76%) patients suffered from low bone mass in the lumbar spine only, while 10 (13.51%) patients had low bone mass in the femoral neck only, and 22 (29.73%) patients had low bone mass in both parts.

In female patients with low bone mass, 27 (54%) had low bone mass in the lumbar region only, 9 (18%) in the femoral neck only, and 14 (28%) had low bone mass in both parts. For male patients with low bone mass, 16 (66.67%) patients had low bone mass only in the lumbar region, and the rest (8, 33.33%) had low bone mass in both parts.

Ten patients had a history of fragility fractures (6 ribs, 3 vertebrae, 1 femoral neck, and ribs), and all of them achieved low bone mass in BMD.

### 3.2. Baseline Characteristics of Cushing's Disease Patients with and without Low Bone Mass

These CD patients were divided into two groups with and without low bone mass (Table 1). Compared to patients without low bone mass, those low bone mass patients had a higher level of diastolic blood pressure (DBP) (97.07 ± 13.69 vs. 89.76 ± 13.43, *p*=0.0016), serum creatinine (66.15 ± 24.33 vs. 55.90 ± 13.35, *p*=0.0026), uric acid (0.36 ± 0.10 vs. 0.32 ± 0.10, *p*=0.0202), cholesterol (5.57 ± 1.30 vs. 5.06 ± 1.47, *p*=0.037), testosterone in women (2.10 (1.33–2.89) vs. 1.54 (0.97–2.05), *p*=0.0012), F24 pm (22.31 (17.95–29.62) vs. 17.80 (13.75–22.77), *p*=0.0006), and higher portion of male (32.43% vs. 11.54%, *p*=0.0016), as well as hypertension (76.12% vs. 51.67%, *p*=0.0075). The low bone mass group had a lower IGF-1 index (0.59 (0.43–0.76) vs. 0.79 (0.60–1.02), *p*=0.0001) and FT3 level (3.54 (3.16–4.04) vs. 3.98 (3.47–4.45), *p*=0.0169) than those without low bone mass. CD patients without low bone mass were more likely to have serum IGF-1 above the upper limit of the normal reference range (ULN) with age-adjusted (18, 26.87% vs. 3, 4.84%, *p*=0.0007). No differences of bone turnover makers were found between the two groups.

### 3.3. Association between Baseline Characteristics and BMD

Spearman's rank correlation analysis was used to explore the related factors of low bone mass in young CD patients (Table 2). The results indicated that the Z score in the lumbar spine was positively associated with age at diagnosis (*r* = 0.18801, *p*=0.0204), IGF-1 index (*r* = 0.35153, *p* < 0.0001), FT3 level (*r* = 0.24117, *p*=0.0055), estradiol in women (*r* = 0.2361, *p*=0.0164), and occurrence of normal menstruation in females (*r* = 0.2267, *p*=0.0136). Meanwhile, SBP (*r* = −0.21575, *p*=0.0099), DBP (*r* = −0.32538, *p* < 0.0001), ALT (*r* = −0.17477, *p*=0.0426), serum creatinine (*r* = −0.36072, *p* < 0.0001), cholesterol (*r* = −0.20205, *p*=0.0197), testosterone in women (*r* = −0.2700, *p*=0.0056), F8 am (*r* = −0.18998, *p*=0.031), and serum cortisol at midnight (*r* = −0.27273, *p*=0.0018) were negatively associated with the Z-score in the lumbar spine. The results also illustrated that the Z-score in the femoral neck was positively associated with BMI (*r* = 0.33926, *p* < 0.0001), IGF-1 index (*r* = 0.24418, *p*=0.0057), FT3 level (*r* = 0.20487, *p*=0.0194), and occurrence of normal menstruation in females (*r* = 0.2393, *p*=0.0094). Serum creatinine (*r* = −0.1932, *p*=0.0248), osteocalcin (*r* = −0.22744, *p*=0.0229**),** and testosterone in women (*r* = −0.2363, *p*=0.0162) were negatively associated with the Z-score in the femoral neck.

### 3.4. IGF-1 Index and Low Bone Mass

**Figure 1:**
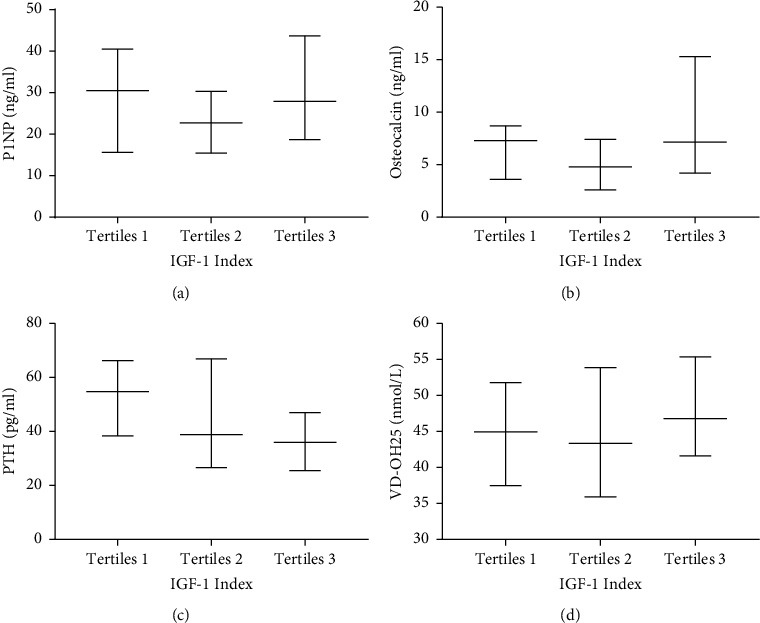
Bone turnover makers in three groups according to tertiles of the preoperative IGF-1 index. Tertiles 1: <0.5986, tertiles 2: 0.5986–0.8380, and tertiles 3 >0.8380. a for PINP; b for osteocalcin; c for PTH; d for VD-OH25. (a) p for trend = 0.2601. (b) p for trend = 0.1310. (c) p for trend = 0.008. (d) p for trend = 0.7956.

**Figure 2:**
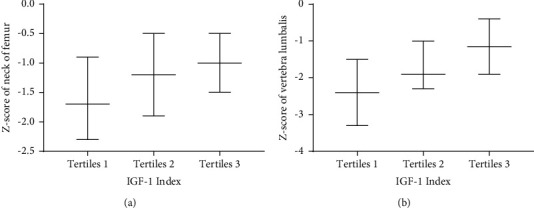
Z-score of both the neck of femur and the vertebra lumbalis in three tertiles of the IGF-1 index. a for the neck of femur; b for the vertebra lumbalis. Tertiles 1: <0.5986, tertiles 2: 0.5986–0.8380, and tertiles 3 >0.8380. (a) p for trend = 0.0148. (b) p for trend < 0.0001.

**Figure 3:**
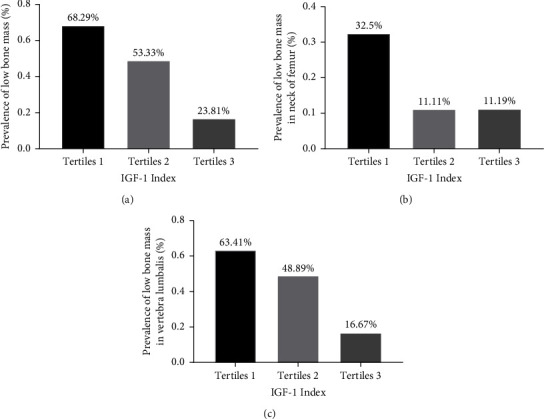
Prevalence of low bone mass according to tertiles of the preoperative IGF-1 index. With increment of the IGF-1 index level, prevalence of low bone mass decreased, both in the vertebra lumbalis and neck of femur. Tertiles 1: <0.5986, tertiles 2: 0.5986–0.8380, and tertiles 3 >0.8380. (a) p for trend = 0.0002. (b) p for trend = 0.0169. (c) p for trend < 0.0001.

Participants were categorized into the following three groups according to tertiles of the preoperative IGF-1 index: <0.5986 (tertiles 1), 0.5986–0.8380 (tertiles 2), and >0.8380 (tertiles 3). With the IGF-1 index increasing, the level of PTH decreased (54.85 (38.35–66.2), 38.9 (26.6–66.9), 36 (25.5–47.05), and *p* = 0.008), while other bone metabolism makers, including PINP, osteocalcin, and 25 (OH) VD, showed no differences among the three groups (Figures 1(a)–1(d)). With the increase in the IGF-1 index level, the Z-score of both vertebra lumbalis (tertiles 1: −2.4 (−3.3∼−1.5); tertiles 2: −1.9 (−2.3∼−1.0); tertiles 3: −1.15 (−1.9∼−0.4), *p* < 0.0001) and the neck of femur (tertiles 1: −1.7 (−2.3∼−0.95); tertiles 2: −1.2 (−1.9∼−0.5); tertiles 3: −1.0 (−1.5∼−0.5), *p* = 0.0148) increased gradually (Figures 2(a) and 2(b)). Meanwhile, prevalence of low bone mass decreased (68.29%, 53.33%, 23.81%, *p* = 0.0002) (Figure 3(a)) both in the vertebra lumbalis (63.41%, 48.89%, 16.67%, *p* < 0.0001) and the neck of femur (32.5%, 11.11%, 11.19%, *p* = 0.0169), with the increasing of the IGF-1 index level (Figures 3(b) and 3(c)).

In the logistic regression analysis of the related factors of low bone mass, most of the potentially relevant factors were put into this model; only the IGF-1 index was still significantly negatively associated with the prevalence of low bone mass after adjusting for covariables. The results indicated that compared to the patients in the lowest tertile of the IGF-1 index (<0.5563), those with the highest tertile of the IGF-1 index (≥0.7993) had a lower prevalence of low bone mass (95% CI 0.16 (0.06–0.41), *p*=0.0002). After adjusting for age, gender, and BMI, the patients in the highest tertile of the IGF-1 index still conferred a lower prevalence of low bone mass (95% CI 0.15 (0.06–0.42), *p*=0.0003). The association between the IGF-1 index and low bone mass still existed (95% CI 0.02 (0.001–0.5), *p*=0.0209) even after adjusting for age, gender, CD duration, BMI, hypertension, dyslipidemia, diabetes, ALT, Scr, FT3, F24 pm, PTH, and osteocalcin (Table 3). In comparison to the reference population, the participants in the middle tertile of the IGF-1 index (0.5563–0.7993) had no different risk of low bone mass.

## 4. Discussion

Our results revealed that low bone mass occurred in around half of young CD patients, affecting more males than females, and mostly in the lumbar spine. The CD patients in our study had a high prevalence (48.37%) of low bone mass at the baseline. This was in accordance with the findings of previous research, and the reported prevalence of osteoporosis due to excess endogenous cortisol ranges from 22% to 59% [19–25]. In this study, CD patients' lumbar vertebrae were more severely affected than the neck of the femur. It is reported that lumbar vertebrae, containing more trabecular bone than femur neck, were more vulnerable to endogenous cortisol [26].

Our results also indicated that men were more prone to low bone mass than women in CD, which was in accordance with several other studies [23, 27, 28]; possibly, the deleterious effect of cortisol excess on BMD might overrule the protective effects of sex hormones, and men were more often hypogonadal compared with women in CD patients. In our study, patients with low bone mass had a significantly higher level of F24 pm. Both cortisol levels in the morning and at midnight, were negatively associated with the Z-score of BMD in the lumbar spine at diagnosis. But these results were not seen in the femoral neck at diagnosis. This further indicated that lumbar vertebrae were more vulnerable to endogenous cortisol. BMI was considered to be associated with bone mass [29]. In our study, higher BMI was associated with higher BMD at diagnosis in the femur neck but not in the lumbar vertebrae, consistent with other studies [30].

Interestingly, besides the above known related factors, we also found that a higher level of the IGF-1 index was strongly associated with a lower prevalence of low bone mass, both in the vertebra lumbalis and the neck of the femur, independently of age, gender, duration, BMI, hypertension, dyslipidemia, diabetes, level of ALT, creatinine, FT3, and F24 pm. The IGF-1 index was also positively associated with the BMD Z-score, both in the lumbar spine and the femoral neck. So far, there have been few studies concerning the association between IGF-1 and low bone mass in Cushing's disease patients. As we know, GH [31, 32] and IGF-1 [33] have been demonstrated to increase both bone formation (e.g., collagen synthesis) and bone resorption. However, in CD patients, glucocorticoids resulted in decreased number and dysfunction of osteoblasts by inhibiting GH-IGF-1 axes [34, 35]. *In vitro* studies suggested that at high concentrations of glucocorticoids, a decreased release of GHRH had been reported [36–38]; therefore, GH-IGF-1 axes were inhibited. IGF-1 possessed anabolic mitogenic actions in osteoblasts while reducing the anabolic actions of TGF-*β* [39]. The decrease in IGF-1 might be a risk factor for low bone mass in CD patients. In vitro studies had also indicated that the suppressive effects of glucocorticoids on osteoblast function can be partially reversed by GH or IGF treatment [8]. In recent years, some studies have also shown that patients with untreated Cushing's disease may have elevated IGF-1, and mildly elevated IGF-1 in Cushing's disease does not imply pathological growth hormone excess. Higher IGF-1 levels could predict better outcomes in CD [40, 41]. Possible mechanisms were not clear, which might involve changes in IGF binding proteins (IGFBPs), interference in IGFBP fragments, IGF-1 synthesis or clearance, and/or the effects of hyperinsulinism induced by excess glucocorticoids. In our study, the results also showed that IGF-1 was an independent protective factor for low bone mass in CD patients.

Our study was one of the few well-powered research studies on the association of IGF-1 levels with low bone mass in young CD patients. These represented important strengths of our study, especially given the rarity of CD. The main limitation of this study was its retrospective nature. This could not prove causality. A prospective study should be conducted to explore the causality between IGF-1 and osteoporosis in CD patients. In addition, this study lacked morphometric data for spinal fractures in all patients, which may underestimate the incidence of fractures and osteoporosis. However, our study indicated that a lower IGF-1 index level was significantly associated with low bone mass in young CD patients, which might provide a new aspect to understand the possible risk factors and mechanism of osteoporosis in CD patients.

In conclusion, our study found that a higher IGF-1 index was independently and significantly associated with decreased prevalence of low bone mass in young CD patients, drawing attention to the role of IGF-1 in the pathogenesis of CD-caused low bone mass and may support the exploration of this pathway in therapeutic agent development in antiosteoporosis in CD.

## Figures and Tables

**Table 1:** Clinical and biochemical preoperative characteristics of young Cushing's disease patients according to status of bone mineral density at diagnosis.

Variables	Without low bone mass	With low bone mass	*p* value
*N* (%)	78 (51.32%)	74 (48.37%)	
Male *N* (%)	9 (11.54%)	24 (32.43%)	0.0016
Female *N* (%)	69 (88.46%)	50 (67.57%)	
History of fragility fractures *N*	0	10	—
Disease status			
Newly diagnosed *N*	59	58	0.6351
Recurrence *N*	13	9	0.1562
No remission after treatment *N*	7	7	0.7453
Osteocalcin (ng/ml)	5.84 (3.60–8.79)	6.71 (3.60–8.90)	0.7267
Decreased osteocalcin *N* (%)	23 (41.07%)	18 (40%)	0.9132
VD-OH25 (nmol/L)	48.45 (37.79–55.85)	44.40 (37.47–53.75)	0.2979
Decreased VD-OH25 *N* (%)	31 (59.62%)	28 (70%)	0.3032
PTH (pg/ml)	35.50 (25.30–54.20)	48.45 (33.70–65.80)	0.1828
Increased PTH *N* (%)	8 (14.04%)	7 (14%)	0.9958
P1NP (ng/ml)	22.97 (14.54–40.21)	30.53 (20.75–41.07)	0.3276
Decreased P1NP *N* (%)	2 (4.35%)	2 (6.06%)	0.7320
Age at diagnosis (year)	34.81 ± 8.19	33.66 ± 8.62	0.4022
Cushing duration (years)	3.11 ± 2.98	3.74 ± 4.47	0.3251
BMI (kg/m^2^)	26.40 ± 5.20	25.24 ± 3.41	0.1228
SBP (mm Hg)	137.13 ± 16.94	142.79 ± 17.64	0.0533
DBP (mm Hg)	89.76 ± 13.43	97.07 ± 13.69	0.0016
HBP *N* %	41 (51.67%)	51 (76.12%)	0.0075
ALT (mmol/L)	26.50 (18.00–42.00)	33.00 (22.00–56.00)	0.0906
Serum creatinine (mmol/L)	55.90 ± 13.35	66.15 ± 24.33	0.0026
UA (mmol/L)	0.32 ± 0.10	0.36 ± 0.10	0.0202
HbA1c %	5.90 (5.50–6.55)	5.75 (5.30–6.50)	0.5194
DM *N* (%)	27 (45.76%)	32 (62.75%)	0.0749
CHOL (mmol/L)	5.06 ± 1.47	5.57 ± 1.30	0.037
TG (mmol/L)	1.32 (0.91–2.13)	1.67 (1.09–2.46)	0.1718
HDL (mmol/L)	1.45 ± 0.42	1.50 ± 0.39	0.4801
LDL (mmol/L)	3.23 ± 0.87	3.48 ± 0.94	0.1125
Dyslipidemia *N* (%)	31 (44.29%)	30 (47.62%)	0.7001
IGF-1 index	0.79 (0.60–1.02)	0.59 (0.43–0.76)	0.0001
IGF-1 index >1 *N* (%)	18 (26.87%)	3 (4.84%)	0.0007
TSH (mIU/L)	1.08 (0.69–1.59)	0.83 (0.59–1.42)	0.1304
FT3 (nmol/L)	3.98 (3.47–4.45)	3.54 (3.16–4.04)	0.0169
FT4 (nmol/L)	13.70 (12.70–15.26)	13.48 (11.59–16.00)	0.6259
Testosterone (ng/L)			
Male	4.47 (3.55–6.47)	6.06 (4.04–9.38)	0.1596
Female	1.54 (0.97–2.05)	2.10 (1.33–2.89)	0.0012
Estradiol (ng/L)			
Male	62.6 (47.5–88.1)	80.25 (38.2–109.3)	0.8229
Female	172.95 (116.25–301.70)	137.50 (89.70–251.50)	0.1754
Normal menstruation in female *N* (%)	35 (50.7%)	16 (32.6%)	0.059
24h UFC (nmol/24 h)	287.28 (172.32–643.40)	345.69 (187.92–722.88)	0.2771
F8 am (ug/dL)	26.18 (17.81–31.96)	28.08 (22.22–33.08)	0.0550
F24 pm (ug/dL)	17.80 (13.75–22.77)	22.31 (17.95–29.62)	0.0006

**Table 2:** Spearman rank correlation of BMD and various variables in Cushing's disease patients.

Variables	Z-score in lumbar spine		Z-score in femoral neck	
	Coefficient	*p* value	Coefficient	*p* value
Age at diagnosis (years)	0.18801	0.0204	0.01793	0.827
Cushing duration (years)	−0.04584	0.5921	−0.15269	0.0738
BMI (kg/m^2^)	0.12494	0.1385	0.33926	<0.0001
SBP (mm Hg)	−0.21575	0.0099	−0.08123	0.3383
DBP (mm Hg)	−0.32538	<0.0001	−0.12527	0.1388
ALT (mmol/L)	−0.17477	0.0426	0.05121	0.5568
AST (mmol/L)	−0.08974	0.3006	−0.00888	0.9189
Serum creatinine (mmol/L)	−0.36072	<0.0001	−0.19319	0.0248
UA (mmol/L)	−0.15829	0.0657	−0.1198	0.1663
HbA1c %	0.12066	0.1715	0.13429	0.1292
Chol (mmol/L)	−0.20205	0.0197	0.01576	0.8577
TG (mmol/L)	−0.16302	0.0608	−0.05957	0.4974
HDL (mmol/L)	−0.14705	0.0912	−0.05434	0.5361
LDL (mmol/L)	−0.15146	0.0818	0.05435	0.5359
IGF-1 index	0.35153	<0.0001	0.24418	0.0057
TSH (mIU/L)	0.11768	0.1807	0.01643	0.8528
Osteocalcin (ng/ml)	−0.07206	0.4739	−0.22744	0.0229
VD-OH25 (nmol/L)	0.11215	0.2871	0.00011	0.9992
PTH (pg/ml)	−0.17208	0.0763	−0.1419	0.1468
P1NP (ng/ml)	−0.11876	0.2972	−0.16851	0.1377
FT3 (nmol/L)	0.24117	0.0055	0.20487	0.0194
FT4 (nmol/L)	0.01175	0.894	0.11381	0.1973
T (ng/L)				
Male	−0.2246	0.2601	−0.1484	0.4602
Female	−0.2700	0.0056	−0.2363	0.0162
Estradiol (ng/L)				
Male	−0.1669	0.4252	0.0392	0.8524
Female	0.2361	0.0164	0.1020	0.3075
Normal menstruation in female *N* (%)	0.2267	0.0136	0.2393	0.0094
24h UF (nmol/24 h)	−0.13934	0.1006	0.02869	0.7375
F8 am (ug/dL)	−0.18998	0.031	−0.14753	0.0966
F24 pm (ug/dL)	−0.27273	0.0018	−0.1455	0.1013

**Table 3:** Association between the preoperative IGF-1 index and the risk of low bone mass.

Tertiles of the IGF-1 index			Adjustment
<0.5563	0.5563∼0.7993	≥0.7993	
Risk of low bone mass OR (95% CI)		
1	0.57 (0.24–1.36)	0.16 (0.06–0.41)	Unadjusted
1	0.61 (0.24–1.55)	0.15 (0.06–0.42)	Adjusted for age, gender, and BMI
1	0.22 (0.03–1.79)	0.02 (0.001–0.50)	Adjusted for age, gender, duration of CS, BMI, hypertension, dyslipidemia, diabetes, ALT, serum creatinine, FT3, F24pm, testosterone, PTH, and osteocalcin

## Data Availability

The data used to support the findings of the study are available on request from the authors.

## Additional Points

Through a retrospective study of a large sample of Cushing's disease (CD) patients from a single center, we found that a higher IGF-1 index was independently associated with a lower prevalence of low bone mass in young CD patients and IGF-1 might play an important role in the pathogenesis of CD-caused low bone mass.
